# Supplementary material for: Vegetation communities on commercial developments are heterogenous and determined by development and landscaping decisions, not socioeconomics
Source: PLoS One. 2019 Sep 10;14(9):e0222069. doi: 10.1371/journal.pone.0222069 (PMC6736242; doi:10.1371/journal.pone.0222069)
Supplement: S1 Table — Abundance is count of individuals belonging to each taxonomic group. Ambiguous indicate both native, non-native, and hybrids used in horticulture. (DOCX) [file pone.0222069.s001.docx]

**S1 Table. All trees observed in site surveys.** Abundance is count of individuals belonging to each taxonomic group. Ambiguous indicate both native, non-native, and hybrids used in horticulture.

| **Taxa** | **Common Name** | **Origin** | **No. Sites Found** | **Abundance Range** | **Total Abundance** | **Mean Abundance** | **Abundance SD** |
| --- | --- | --- | --- | --- | --- | --- | --- |
| *Acer ginnala* | Amur maple | Non-native | 1 | 0–2 | 2 | 0.10 | 0.45 |
| *Acer macrophyllum* | Bigleaf maple | Native | 11 | 0–47 | 218 | 10.90 | 17.15 |
| *Acer platanoides* | Norway maple | Non-native | 6 | 0–50 | 100 | 5.00 | 12.07 |
| *Acer rubrum* | Red maple | Non-native | 9 | 0–33 | 132 | 6.60 | 9.72 |
| *Acer saccharum* | Sugar maple | Non-native | 3 | 0–24 | 43 | 2.15 | 6.52 |
| *Alnus rubra* | Red alder | Native | 9 | 0–7 | 29 | 1.45 | 2.31 |
| *Arbutus menziesii* | Pacific madrone | Native | 6 | 0–12 | 19 | 0.95 | 2.68 |
| *Betula occidentalis* | Red birch | Non-native | 1 | 0–3 | 3 | 0.15 | 0.67 |
| *Betula papyrifera* | Paper birch | Native | 4 | 0–2 | 7 | 0.35 | 0.74 |
| *Betula pendula* | Silver birch | Non-native | 2 | 0–2 | 3 | 0.15 | 0.49 |
| *Carpinus* | Hornbeam | Non-native | 1 | 0–3 | 3 | 0.15 | 0.67 |
| *Cedrus deodara* | Deodar cedar | Non-native | 4 | 0–4 | 13 | 0.65 | 1.39 |
| *Cercidiphyllum japonicum* | Katsura | Non-native | 1 | 0–5 | 5 | 0.25 | 1.12 |
| *Cercis canadensis* | Redbud | Non-native | 2 | 0–6 | 8 | 0.40 | 1.39 |
| *Cupressus nootkatensis* | Alaska cedar | Native | 4 | 0–39 | 57 | 2.85 | 8.91 |
| *Fagus sylvatica gp.* | Beech | Non-native | 2 | 0–1 | 2 | 0.10 | 0.31 |
| *Fraxinus americana* | White ash | Non-native | 2 | 0–26 | 35 | 1.75 | 6.05 |
| *Fraxinus pennsylvanica* | Green ash | Non-native | 2 | 0–16 | 17 | 0.85 | 3.57 |
| *Gleditsia triacanthos* | Honey locust | Non-native | 2 | 0–8 | 9 | 0.45 | 1.79 |
| *Liquidambar styraciflua* | Sweetgum | Non-native | 3 | 0–11 | 24 | 1.20 | 3.16 |
| *Liriodendron tulipifera* | Tulip tree | Non-native | 3 | 0–4 | 7 | 0.35 | 0.99 |
| *Magnolia grandiflora* | Southern magnolia | Non-native | 3 | 0–5 | 11 | 0.55 | 1.43 |
| *Malus* | Apple | Non-native | 3 | 0–4 | 6 | 0.30 | 0.92 |
| *Picea omorika* | Weeping Serbian spruce | Non-native | 1 | 0–6 | 6 | 0.30 | 1.34 |
| *Pinus contorta* | Shore pine | Native | 1 | 0–8 | 8 | 0.40 | 1.79 |
| *Pinus nigra* | Black pine | Non-native | 5 | 0–42 | 75 | 3.75 | 10.94 |
| *Pinus strobus* | White pine | Ambiguous | 1 | 0–1 | 1 | 0.05 | 0.22 |
| *Pinus sylvestris* | Scots pine | Non-native | 4 | 0–7 | 17 | 0.85 | 2.06 |
| *Platanus occidentalis* | American sycamore | Non-native | 2 | 0–2 | 3 | 0.15 | 0.49 |
| *Populus nigra* | Lombardy poplar | Non-native | 1 | 0–9 | 9 | 0.45 | 2.01 |
| *Populus tremuloides* | Quaking aspen | Non-native | 1 | 0–17 | 17 | 0.85 | 3.80 |
| *Populus trichocarpa* | Black cottonwood | Native | 6 | 0–4 | 13 | 0.65 | 1.27 |
| *Prunus* | Flowering plum or cherry | Non-native | 12 | 0–9 | 39 | 1.95 | 2.82 |
| *Prunus subg. Padus* | Choke cherries | Non-native | 1 | 0–2 | 2 | 0.10 | 0.45 |
| *Pseudotsuga menziesii* | Douglas fir | Native | 18 | 0–204 | 746 | 37.30 | 50.75 |
| *Quercus palustris* | Pin oak | Non-native | 2 | 0–4 | 8 | 0.40 | 1.23 |
| *Quercus rubra* | Red oak | Non-native | 2 | 0–9 | 11 | 0.55 | 2.04 |
| *Robinia pseudoacacia* | Black locust | Non-native | 1 | 0–3 | 3 | 0.15 | 0.67 |
| *Sorbus aucuparia* | European mountain ash | Non-native | 1 | 0–1 | 1 | 0.05 | 0.22 |
| *Taxus brevifolia* | Pacific yew | Native | 1 | 0–1 | 1 | 0.05 | 0.22 |
| *Thuja plicata* | Western red cedar | Native | 15 | 0–53 | 245 | 12.25 | 15.27 |
| *Tilia* | Linden | Non-native | 1 | 0–1 | 1 | 0.05 | 0.22 |
| *Tsuga heterophylla* | Western hemlock | Native | 3 | 0–3 | 5 | 0.25 | 0.72 |
| *Ulmu* | Elm | Non-native | 1 | 0–4 | 4 | 0.20 | 0.89 |
| Broadleaf 1 |  | Ambiguous | 1 | 0–2 | 2 | 0.10 | 0.45 |
| Broadleaf 2 |  | Ambiguous | 1 | 0–1 | 1 | 0.05 | 0.22 |
| Broadleaf 3 |  | Ambiguous | 1 | 0–1 | 1 | 0.05 | 0.22 |
| Broadleaf 4 |  | Ambiguous | 1 | 0–1 | 1 | 0.05 | 0.22 |
| Broadleaf 5 |  | Ambiguous | 1 | 0–2 | 2 | 0.10 | 0.45 |
| Broadleaf 6 |  | Ambiguous | 1 | 0–1 | 1 | 0.05 | 0.22 |
| Cedar 1 |  | Ambiguous | 1 | 0–1 | 1 | 0.05 | 0.22 |
| Cedar 2 |  | Ambiguous | 1 | 0–1 | 1 | 0.05 | 0.22 |
