# Supplementary material for: Vegetation communities on commercial developments are heterogenous and determined by development and landscaping decisions, not socioeconomics
Source: PLoS One. 2019 Sep 10;14(9):e0222069. doi: 10.1371/journal.pone.0222069 (PMC6736242; doi:10.1371/journal.pone.0222069)
Supplement: S2 Table — Abundance is count of individuals belonging to each taxonomic group. Ambiguous indicate both native, non-native, and hybrids used in horticulture. (DOCX) [file pone.0222069.s002.docx]

**S2 Table. All shrubs observed in site surveys.** Abundance is count of individuals belonging to each taxonomic group. Ambiguous indicate both native, non-native, and hybrids used in horticulture.

| **Taxa** | **Common Name** | **Origin** | **No. Sites Found** | **Abundance Range** | **Total Abundance** | **Mean Abundance** | **Abundance SD** |
| --- | --- | --- | --- | --- | --- | --- | --- |
| *Abelia grandiflora* | Glossy abelia | Non-native | 1 | 0–29 | 29 | 1.45 | 6.485 |
| *Acer circinatum* | Vine maple | Native | 13 | 0–75 | 265 | 13.25 | 19.628 |
| *Acer palmatum* | Japanese maple | Non-native | 4 | 0–9 | 17 | 0.85 | 2.183 |
| *Amelanchier alnifolia* | Saskatoon | Native | 1 | 0–4 | 4 | 0.20 | 0.894 |
| *Arbutus unedo* | Strawberry tree | Non-native | 5 | 0–63 | 125 | 6.25 | 17.262 |
| *Arctostaphylos* | Manzanita | Native | 1 | 0–15 | 15 | 0.75 | 3.354 |
| *Aucuba japonica* | Spotted laurel | Non-native | 3 | 0–4 | 7 | 0.35 | 0.988 |
| *Mahonia gp.* | Mahonia gp. | Ambiguous | 16 | 0–359 | 1,007 | 50.35 | 93.443 |
| *Berberis thunbergii* | Barberry | Non-native | 5 | 0–93 | 144 | 7.20 | 21.222 |
| *Buxus* | Boxwood | Non-native | 6 | 0–52 | 65 | 3.25 | 11.548 |
| *Calluna vulgaris* | Common heather | Non-native | 3 | 0–25 | 28 | 1.40 | 5.576 |
| *Cistaceae* | Rock rose | Non-native | 2 | 0–39 | 56 | 2.80 | 9.328 |
| *Cornus florida gp.* | Flowering dogwood gp. | Non-native | 4 | 0–6 | 12 | 0.60 | 1.569 |
| *Cornus sericea gp.* | Tatarian dogwood | Non-native | 10 | 0–162 | 419 | 20.95 | 43.685 |
| *Corylus* | Hazelnut | Native | 9 | 0–58 | 109 | 5.45 | 13.249 |
| *Cotoneaster* | Cotoneaster | Non-native | 8 | 0–39 | 85 | 4.25 | 9.296 |
| *Crataegus* | Hawthorn | Ambiguous | 5 | 0–4 | 11 | 0.55 | 1.146 |
| *Cytisus scoparius* | Scotch broom | Non-native | 3 | 0–6 | 9 | 0.45 | 1.395 |
| *Daphne* | Daphne | Non-native | 1 | 0–4 | 4 | 0.20 | 0.894 |
| *Dasiphora fruticosa* | White potentilla | Native | 1 | 0–19 | 19 | 0.95 | 4.249 |
| *Elaeagnus commutata* | Silverberry | Native | 1 | 0–31 | 31 | 1.55 | 6.932 |
| *Enkianthus campanulatus* | Enkianthus | Non-native | 1 | 0–2 | 2 | 0.10 | 0.447 |
| *Erica* | Winter heath | Non-native | 5 | 0–6 | 16 | 0.80 | 1.609 |
| *Escallonia* | Escallonia | Non-native | 3 | 0–17 | 40 | 2.00 | 5.301 |
| *Euonymus alatus* | Winged euonymus | Non-native | 4 | 0–41 | 127 | 6.35 | 13.620 |
| *Euonymus japonicus* | Japanese spindle | Non-native | 9 | 0–15 | 76 | 3.80 | 5.197 |
| *Euphorbia* | Euphorbia | Non-native | 5 | 0–21 | 56 | 2.80 | 5.662 |
| *Forsythia sp.* | Forsythia | Non-native | 4 | 0–63 | 76 | 3.80 | 14.059 |
| *Gardenia sp.* | Gardenia | Non-native | 1 | 0–3 | 3 | 0.15 | 0.671 |
| *Gaultheria shallon* | Salal | Native | 13 | 0–393 | 1,267 | 63.35 | 115.281 |
| *Hebe sp.* | Hebe | Non-native | 2 | 0–21 | 31 | 1.55 | 5.094 |
| *Hibiscus syriacus* | Rose of Sharon (hibiscus) | Non-native | 1 | 0–2 | 2 | 0.10 | 0.447 |
| *Holodiscus discolor* | Oceanspray | Native | 1 | 0–7 | 7 | 0.35 | 1.565 |
| *Hydrangea sp.* | Hydrangea | Non-native | 4 | 0–6 | 15 | 0.75 | 1.860 |
| *Hypericum calycinum* | St. John’s wort | Non-native | 3 | 0–29 | 71 | 3.55 | 8.894 |
| *Ilex aquifolium gp.* | English holly group | Non-native | 8 | 0–11 | 30 | 1.50 | 2.947 |
| *Ilex crenata* | Japanese holly | Non-native | 8 | 0–29 | 138 | 6.90 | 10.711 |
| *Kalmia latifolia* | Mountain laurel | Non-native | 1 | 0–2 | 2 | 0.10 | 0.447 |
| *Lavandula sp.* | Lavender | Non-native | 3 | 0–43 | 46 | 2.30 | 9.592 |
| *Leucothoe fontanesiana* | Drooping Leucothoe | Non-native | 2 | 0–5 | 8 | 0.40 | 1.273 |
| *Leycesteria formosa* | Himalayan honeysuckle | Non-native | 1 | 0–17 | 17 | 0.85 | 3.801 |
| *Lonicera pileata* | Privet honeysuckle | Non-native | 1 | 0–136 | 136 | 6.80 | 30.411 |
| *Lonicera sempervirens gp.* | Trumpet honeysuckle | Non-native | 1 | 0–9 | 9 | 0.45 | 2.012 |
| *Nandina domestica* | Heavenly bamboo | Non-native | 12 | 0–106 | 168 | 8.40 | 23.359 |
| *Oemleria cerasiformis* | Indian plum | Native | 9 | 0–44 | 146 | 7.30 | 11.721 |
| *Ornamental conifer* | Ornamental conifer | Non-native | 13 | 0–40 | 177 | 8.85 | 11.431 |
| *Osmanthus x burkwoodii gp.* | Burkwood osmanthus | Non-native | 3 | 0–64 | 76 | 3.80 | 14.348 |
| *Philadelphus lewisii* | Mock orange | Native | 1 | 0–2 | 2 | 0.10 | 0.447 |
| *Photinia x fraseri gp.* | Fraser’s Photinia | Non-native | 7 | 0–22 | 66 | 3.30 | 6.131 |
| *Physocarpus opulifolius* | Ninebark | Non-native | 1 | 0–9 | 9 | 0.45 | 2.012 |
| *Pieris japonica* | Japanese pieris | Non-native | 6 | 0–11 | 34 | 1.70 | 3.310 |
| *Prunus laurocerasus* | Cherry laurel | Non-native | 16 | 0–145 | 682 | 34.10 | 39.202 |
| *Rhaphiolepis indica* | Indian hawthorne | Non-native | 1 | 0–4 | 4 | 0.20 | 0.894 |
| *Rhododendron sp.* | Rhododendron | Non-native | 20 | 4–76 | 612 | 30.60 | 21.507 |
| *Rhus sp.* | Sumac | Non-native | 3 | 0–18 | 25 | 1.25 | 4.115 |
| *Ribes sanguineum* | Red flowering currant | Native | 6 | 0–47 | 143 | 7.15 | 14.057 |
| *Rosa sp.* | Domestic rose | Non-native | 3 | 0–26 | 46 | 2.30 | 6.760 |
| *Rosmarinus officinalis* | Rosemary | Non-native | 1 | 0–1 | 1 | 0.05 | 0.224 |
| *Rubus bifrons* | Himalayan blackberry | Non-native | 7 | 0–35 | 119 | 5.95 | 10.655 |
| *Rubus laciniatus* | Evergreen blackberry | Non-native | 1 | 0–25 | 25 | 1.25 | 5.590 |
| *Rubus spectabilis* | Salmonberry | Native | 3 | 0–25 | 48 | 2.40 | 6.954 |
| *Sambucus sp.* | Elderberry | Ambiguous | 1 | 0–3 | 3 | 0.15 | 0.671 |
| *Sarcococca confusa* | Sweet box | Non-native | 4 | 0–51 | 69 | 3.45 | 11.528 |
| *Spiraea japonica gp.* | Spiraea japonica gp. | Non-native | 2 | 0–51 | 57 | 2.85 | 11.412 |
| *Spiraea nipponica* | Japanese spirea | Non-native | 2 | 0–52 | 59 | 2.95 | 11.651 |
| *Styrax japonicus* | Japanese snowball | Non-native | 4 | 0–5 | 11 | 0.55 | 1.317 |
| *Symphoricarpos sp.* | Snowberry | Native | 8 | 0–58 | 148 | 7.40 | 14.848 |
| *Syringa vulgaris* | Lilac | Non-native | 2 | 0–1 | 2 | 0.10 | 0.308 |
| *Thuja occidentalis* | Arborvitae | Non-native | 4 | 0–14 | 31 | 1.55 | 4.006 |
| *Vaccinium ovatum* | Evergreen huckleberry | Native | 6 | 0–155 | 205 | 10.25 | 34.727 |
| *Vaccinium parvifolium* | Huckleberry | Native | 6 | 0–7 | 26 | 1.30 | 2.273 |
| *Vaccinium sect. Cyanococcus* | Blueberry | Native | 1 | 0–5 | 5 | 0.25 | 1.118 |
| *Viburnum davidii* | David viburnum | Non-native | 8 | 0–69 | 278 | 13.90 | 22.525 |
| *Viburnum plicatum* | Snowball bush | Non-native | 2 | 0–6 | 11 | 0.55 | 1.701 |
| *Viburnum tinus* | Laurustinus | Non-native | 8 | 0–31 | 101 | 5.05 | 9.644 |
| Unknown B gp. |  | Ambiguous | 1 | 0–1 | 1 | 0.05 | 0.224 |
| Unknown C gp. |  | Ambiguous | 1 | 0–2 | 2 | 0.10 | 0.447 |
| Unknown F gp. |  | Ambiguous | 1 | 0–1 | 1 | 0.05 | 0.224 |
| Unknown G gp. |  | Ambiguous | 1 | 0–1 | 1 | 0.05 | 0.224 |
| Unknown J gp. |  | Ambiguous | 1 | 0–1 | 1 | 0.05 | 0.224 |
| Unknown K gp. |  | Ambiguous | 1 | 0–1 | 1 | 0.05 | 0.224 |
| Unknown L gp. |  | Ambiguous | 1 | 0–1 | 1 | 0.05 | 0.224 |
| Unknown N gp. |  | Ambiguous | 1 | 0–5 | 5 | 0.25 | 1.118 |
| Unknown S gp. |  | Ambiguous | 1 | 0–1 | 1 | 0.05 | 0.224 |
