# Supplementary material for: Vegetation communities on commercial developments are heterogenous and determined by development and landscaping decisions, not socioeconomics
Source: PLoS One. 2019 Sep 10;14(9):e0222069. doi: 10.1371/journal.pone.0222069 (PMC6736242; doi:10.1371/journal.pone.0222069)
Supplement: S3 Table — (DOCX) [file pone.0222069.s003.docx]

**S3 Table. All simple multivariate PERMANOVA results for tree and shrub communities.** Variables significant at α ≤ 0.05 after Holm-Bonferroni correction for multiple comparisons are bolded. For trees, median household income is only significant at α ≤ 0.1 after multiple comparison correction.

| **Model** | **Shrub Pseudo-*F*** | **Shrub  p-value** | **Adjusted Shrub p-value** | **Shrub AICc** | **Tree Pseudo-*F*** | **Tree  p-value** | **Adjusted Tree p-value** | **Tree AICc** |
| --- | --- | --- | --- | --- | --- | --- | --- | --- |
| Area (acres) | 1.64 | 0.060 | 0.660 | 36.6 | 1.07 | 0.377 | 1.00 | 37.4 |
| Foreign-Born (%) | 1.50 | 0.093 | 0.910 | 36.7 | 0.69 | 0.707 | 1.00 | 37.8 |
| Town | 1.16 | 0.268 | 1.00 | 37.1 | 0.84 | 0.535 | 1.00 | 37.6 |
| Median Household Income (USD) | 1.35 | 0.157 | 1.00 | 36.9 | 2.62 | 0.008 | 0.088 | 35.8 |
| Impervious w/in 500 m (%) | 0.87 | 0.572 | 1.00 | 37.4 | 0.84 | 0.553 | 1.00 | 37.6 |
| Building Age (years in 2017) | 1.55 | 0.091 | 0.910 | 36.7 | 0.89 | 0.529 | 1.00 | 37.5 |
| Building Quality | 1.14 | 0.253 | 1.00 | 40.4 | 1.44 | 0.112 | 0.896 | 39.7 |
| Appraised Land Value per Acre (USD) | 1.18 | 0.269 | 1.00 | 37.0 | 0.48 | 0.900 | 1.00 | 38.0 |
| **Tree Cluster (Native/Orn.)** | **2.86** | **0.001** | **0.015** | **35.4** |  |  |  |  |
| Dead Wood (count) | 1.48 | 0.128 | 1.00 | 36.7 |  |  |  |  |
| **Median Height of Dominant Conifer (m)** | **3.08** | **0.002** | **0.028** | **35.1** |  |  |  |  |
| Stands Predate Development | 2.26 | 0.013 | 0.156 | 35.9 |  |  |  |  |
| **Density of Native Conifers (count/acre)** | **2.82** | **0.003** | **0.039** | **35.4** |  |  |  |  |
