# Supplementary material for: Vegetation communities on commercial developments are heterogenous and determined by development and landscaping decisions, not socioeconomics
Source: PLoS One. 2019 Sep 10;14(9):e0222069. doi: 10.1371/journal.pone.0222069 (PMC6736242; doi:10.1371/journal.pone.0222069)
Supplement: Attachment — Submitted filename: Response to Reviewers.docx [file pone.0222069.s004.docx]

Karen Dyson
Research and Design for Integrated Ecology
Urban Ecology Research Laboratory
Seattle, WA, USA

Dr. Zhihua Wang
Academic Editor
PLOS ONE

Dear Dr. Wang,

Thank you for the opportunity to revise my manuscript (PONE-D-19-17662) previously titled “Heterogeneity within and between land uses: commercial office vegetation is determined by development and landscaping decisions.”

I appreciate the reviewer’s comments and have found them helpful in guiding my revisions. In response to reviewer’s concerns, I have revised the title and text of the manuscript, as well as Figures 2 and 5. Below please find detailed responses.

I hope the revised manuscript will better suit PLOS ONE, but I am happy to consider further revisions. I thank you for your interest in my research.

Best wishes,

Karen Dyson

**Reviewer 1:**

**1. Figure 2: it would be good to colour each vegetation type for better presentation of the heterogeneity; meanwhile the sites sampled can be indicated with symbols different from circles.**

**2. Figure 5: I doubt a line plot would hinder the interpretation of the underlying distribution: a scatter plot with vegetation types coloured might be more informative.**

- I have revised Figure 2 so that each vegetation type is a different color, using a color-blind and printer-friendly color scheme. I have also used different shapes, as suggested.
- I have also revised Figure 5 so that each vegetation type is distinct, using the same color scheme as Figure 2. I used a stacked histogram to show the distribution and each vegetation types’ contribution.

**Reviewer 2:**

**First of all, the title of this manuscript is too long without clear representation of the context. I see that the author tried to link the current findings on commercial office developments to the previous findings on residential areas. Specifically, the key factor of residential area is the socio-economic factor, while the key factors of commercial area are the development & landscaping decisions. However, the manuscript is mainly describing and discussing the data collected in commercial office development areas. Therefore, the title “Heterogeneity within and between land uses” is not appropriate. I would argue that this is only heterogeneity within the commercial land use.**

**The methodology is not clearly described. For example, the main statistical model PERMANOVA and the non-metric multidimensional scaling (NMDS) method are not well explained before using. A lot of abbreviations like ANOVA and AICc function are not described.**

**In the section of independent variables, an overview of independent variables should be presented at first. For example, you could mention at the beginning that three categories of independent variables are collected, including 1. aggregated and parcel level socio-economic variables, 2. development and landscaping outcome variables, 3. ground cover material and maintenance regime variables.**

**Can you define aggregated level and parcel level?**

**Line 49. What is a Puget Sound Region?**

**Line 214, what is "agnes" function?**

**Line 419, you mentioned that "the choice of sampling design and statistical method can result in inaccurate conclusions". Then can you explain how you chose the sampling design and statistical method in this manuscript?**

**Fig 1 & 4 are not referred anywhere in the context.**

**Fig 2. Population legend, please add the abbreviations HH for High, MC for Medium Canopy, etc. here since the abbreviations are displayed in the figure.**

**Some grammar errors exist.**

- I have changed the title to reflect my two key conclusions, specifically heterogeneity within land use and importance of development and landscaping decision over socio-economic factors, as noted by reviewer. I have eliminated reference to heterogeneity between land uses, as suggested.
- I have revised the methods section for added clarity, including general language revisions, adding un-abbreviated names for ANOVA and AICc, adding an explanation of PERMANOVA, and adding an explanation of NMDS.
- I added additional text to the beginning of the ‘Independent variables’ section for clarity. I also introduce Table 2, which presents an overview of independent variables.
- For additional clarity, I added the definition of aggregated scale socio-economic variables to the start of independent variable section (in addition to existing discussion in the Introduction) and revised language throughout the manuscript. I also added a definition of parcel level to the independent variable section.
- Line 49: I added additional description of the Puget Sound region.
- Line 214: I added clarifying text about the agnes function
- Line 419: I added multiple sentences clarifying the need to sample across key gradients to obtain accurate results, as I did here.
- I added a reference to Fig 1. Fig 4 was referenced in line 367.
- I added the abbreviations for vegetation types to the caption of Fig 2 to aid with interpretation, as helpfully suggested.
- I found and corrected multiple grammar errors.

**Journal requirements:**

- Reviewed style templates and altered section headings, placement of Fig and Table captions, and file names in response.
- Added references to Fig 1 and Table 2.
